# Evidence of increasing incidence of type 1 diabetes and ketoacidosis among children in the Republic of Srpska in period 2017–2022 with special focus on COVID-19 global pandemic years

**DOI:** 10.3389/fpubh.2023.1264099

**Published:** 2023-09-06

**Authors:** Gordana Bukara-Radujkovic, Vesna Miljkovic, Olivera Ljuboja

**Affiliations:** ^1^Pediatric Clinic, University Clinical Center of the Republic of Srpska, Banja Luka, Bosnia and Herzegovina; ^2^Department of Pediatric, Faculty of Medicine, University of Banja Luka, Banja Luka, Bosnia and Herzegovina

**Keywords:** type 1 diabetes mellitus, COVID-19, incidence, ketoacidosis, children

## Abstract

**Background and objectives:**

Primary focus of the research was to determine the incidence of type 1 diabetes mellitus in the period from 2017 to 2022, and whether COVID-19 had an impact on the increase in the number of newly diagnosed children with diabetes type 1 under the age of 15 in the Republic of Srpska (Bosnia and Herzegovina). In the period 2001–2016 the incidence of type 1 diabetes mellitus was 11/100,000, with an annual increasing rate of 14.2%.

**Methods:**

Available data from pediatric endocrinology clinics, in the Republic of Srpska, on the number of newly diagnosed patients with diabetes mellitus in the period from January 1, 2017 until December 31, 2022 were used. A retrospective analysis was performed, and the capture-recapture method was used for the final assessment, and the obtained result corresponds to about 99% of the population.

**Results:**

The total number of children in the group of 0–14 years of age diagnosed with type 1 diabetes mellitus in this period was 183, of which 96 (52.46%) were boys, and 87 (47.54%) were girls. The average age at which diabetes mellitus was diagnosed was 8.3 ± 3.9 years. Average incidence of diabetes in the period 2017–2022 was 19/100,000 (95% CI 13.1–25.0). The highest incidence was 28.7/100,000 in 2020, the first year of the global COVID-19 pandemic. Out of a total of 183 newly diagnosed cases in the period 2017–2022, 73 (39.9%) were diagnosed with ketoacidosis upon admission. The largest number of newly diagnosed children was recorded in the group of children aged 10–14 years.

**Conclusion:**

In the last 6 years, there has been a significant increase in the incidence of type 1 diabetes mellitus in children under the age of 15. With an incidence of 19.4/100,000 in the Republic of Srpska, we entered the group of countries with high-risk for diabetes. Further steps must focus on the education of the entire society in order to recognize the symptoms of the disease in time and prevent the occurrence of ketoacidosis, which could significantly reduce the burden on health systems, especially in times of global pandemics, such as the COVID-19 pandemic.

## 1. Introduction

Diabetes mellitus type 1 is a chronic disease defined by a complete lack of insulin caused by the autoimmune destruction of pancreatic beta cells, which leads to an increase in glycaemia. This requires lifelong insulin replacement and lifestyle changes with regular glycemic control (1). It is also one of the most common endocrine and metabolic diseases in childhood (2).

In 2021, the number of newly discovered cases of diabetes mellitus in children under the age of 19 was 355,900 (95% CI: 334,200–377,300), and estimation indicate that by 2050, this number could be higher for 100,000 children per year (3). Unfortunately, this unfavorable trend also reflects on our country. The previous study of type 1 diabetes mellitus incidence in the Republic of Srpska for the period from 2001 to 2016, showed that the incidence was 11/100,000 children, which put us in the group of countries with a medium risk for the disease (4). In that study, the largest number of patients with diabetes mellitus type 1 was in the group of children aged 10–14, unlike most countries where the largest number of patients were in the group of children under 5 years of age, which was explained by the “accelerator hypothesis” (5).

Continuing the previous research, we wanted to see if the growth trend continued in the coming period from 2017 to 2022. Compared to earlier research, this research is specific because it covered the entire period of the COVID-19 infection and it was interesting to see the possible impact of COVID-19 on the number of children with type 1 diabetes mellitus.

On March 11, 2020 the World Health Organization (WHO) declared a global pandemic caused by one strain of coronavirus—SARS CoV-2, calling it COVID-19. The first published studies suggested that children tolerated infection better than adults (6, 7). However, in an effort to prevent the spread and emergence of new cases of this infection in the Republic of Srpska, as in most countries of the world, restrictive measures (isolation, home learning, ban on extracurricular and sports activities) were adopted during 2020 and 2021, which included citizens of all age, especially children. The incidence of diabetes mellitus in children aged 0–14 years in the period of the previous 6 years is extremely interesting and significant data, because it tells us not only about the potential impact of COVID-19 on the occurrence of diabetes (8), but also about the impact of the restrictive measures. Compared to 2019, the estimated incidence in 2021 according to the International Diabetes Federation (IDF) has increased (9), which indicates that globally there was an increase in newly discovered cases of diabetes mellitus in the period of 2020, the first year of the global COVID-19 pandemic.

The potential increase in the number of newly discovered cases of diabetes mellitus during the first year of the global pandemic can be explained by the mechanism of interaction of the virus with the target cell or, more precisely, the receptor site to which the virus binds. Angiotensin-converting enzyme 2 (ACE2) receptor is the binding site of SARS-CoV-1 and-2 viruses and these receptors are strongly expressed in pancreatic cells, and initial findings hypothesized that the SARS-CoV-1 virus enters pancreatic cells via the ACE2 receptor and leads to the destruction of β-cells and, consequently, the appearance of a diabetes mellitus (10). Although most studies did not examine direct exposure to the SARS-CoV-2 virus and the consequent occurrence of diabetes, it is assumed that the infection itself leads to an increase in the number of newly diagnosed type 1 diabetes, accelerating the onset of autoantibodies that destroy β-cells (11). Children initially had milder symptoms of the virus infection, often with negative tests, which were not highly specific and internationally standardized, so there is a possibility that they were exposed to the virus itself, but unrecognized. Also, subsequent serological analyzes were not performed, which could confirm or reject this hypothesis.

Studies investigating the incidence of type 1 diabetes during the COVID-19 pandemic have shown mixed results. An analysis in London found an increase in newly diagnosed type 1 in the state of severe ketoacidosis with pronounced hypokalemia between March 23, 2020 and June 4, 2020, compared to the number of patients in the previous 5 years (11). In Canada during 2020, there was no significant increase in number of newly diagnosed patients comparing to 2019, but the number of ketoacidosis was significantly increased (12). In Germany, in the initial months of the COVID-19 infection (March, April, May 2020), the number of patients was lower compared to previous years, which was explained by restrictive measures and isolation that reduced exposure of infection of not just Sars-CoV-2 virus but, also, of the other common viruses in the pediatric population. However, the appearance of newly diagnosed patients with type 1 diabetes did not stop, which is explained by the fact that isolation led to the stress as a possible trigger for the onset of type 1 diabetes (13).

The most common complication of untreated diabetes mellitus in children is diabetic ketoacidosis, which can lead to death (1). Taking all of this into account, in this work we wanted to determine incidence of type 1 diabetes mellitus from 2017 to 2022, as well as the number of children with ketoacidosis. We will also determine whether there is a connection with the increased number of patients and the frequency of ketoacidosis in the period of 2020 and 2021, the period of COVID-19 global pandemic.

## 2. Materials and methods

The research covered the population of the Republic of Srpska from 0 to 14 years of age in the period 2017–2022. Republic of Srpska is an autonomous part of Bosnia and Herzegovina, which is located in the southeastern part of Europe, and according to data from the last census, it had 1,171,179 people. The last population census of Bosnia and Herzegovina was carried out in 2013, and since then the Republic Institute of Statistics of the Republic of Srpska estimates the number of people on the basis of demographic parameters every year and published the results in its yearbooks, and this data, as the only official one, were used in this research (14).

As there is no official register of patients with diabetes mellitus in the Republic of Srpska, yet, we used available data from pediatric endocrinology clinics in the country on the number of newly diagnosed patients with diabetes mellitus type 1 in the period from January 1, 2017 until December 31, 2022. Data were cross-referenced with official data from the Health Insurance Fund of the Republic of Srpska, which provides free insulin and blood sugar strips to patients up to 15 years of age. The capture-recapture method was used for the final assessment, and the obtained result corresponds to approximately 99% of the population. The criteria that the patients had to fulfill were: that they were diagnosed with type 1 diabetes in the period 2017–2022 by a doctor at one of the pediatric endocrinology clinics in the Republic of Srpska, that they were citizens of the Republic of Srpska at the time of diagnosis, and that they had between 0 and 14 years at the time of diagnosis. These criteria were taken from the EURODIAB study (15).

### 2.1. Statistical analysis

The incidence was calculated as the number of children diagnosed with type one diabetes mellitus per 100,000 children of the same age. The number of children of the same age was calculated based on the number that was on the census in 2013 and the ratio of the total number of people on the census to the estimated total number of people for each year according to the data from the Statistical Yearbook of the Republic Institute of Statistics of the Republic of Srpska. Numerical variables were examined based on measures of central distribution and variability, and categorical variables based on percentages and frequencies. Various tests were used in further statistical analysis, which will be presented in the results.

## 3. Results

One hundred and eighty three children in total were diagnosed with type 1 diabetes mellitus in period 2017–2022. 96 (52.46%) of them were boys, and 87 (47.54%) were girls. The average age at which diabetes mellitus was diagnosed was 8.3 ± 3.9 years.

As it can be seen from Table 1, in the period between 2017 and 2022, the incidence of diabetes mellitus in children aged 0–14 years was 19/100,000/yr., with a 95% confidence interval between 13.1 and 25.0 children (Poisson distribution). The highest incidence was established in 2020 at 28.7/100,000, and the lowest at 12.9/100,000 in 2017. After 2020 the incidence of type one diabetes is in a slight decline.

**Table 1:** Incidence of type 1 diabetes mellitus per 100,000 children per year aged 0–14 in total and according to gender in the period from 2017 to 2022.

Year	Number of newly diagnosed children			Incidence per 100,000 children		
	All	Boys	Girls	All	Boys	Girls
2017	21	14	7	12.9	16.8	8.8
2018	25	13	12	15.5	15.7	15.3
2019	36	14	22	22.4	17.0	28.1
2020	46	22	24	28.7	26.8	30.8
2021	28	19	9	17.6	23.3	11.6
2022	27	14	13	17.0	17.2	16.8
Total	183	96	87	19.0	19.5	18.6

**Figure 1:**
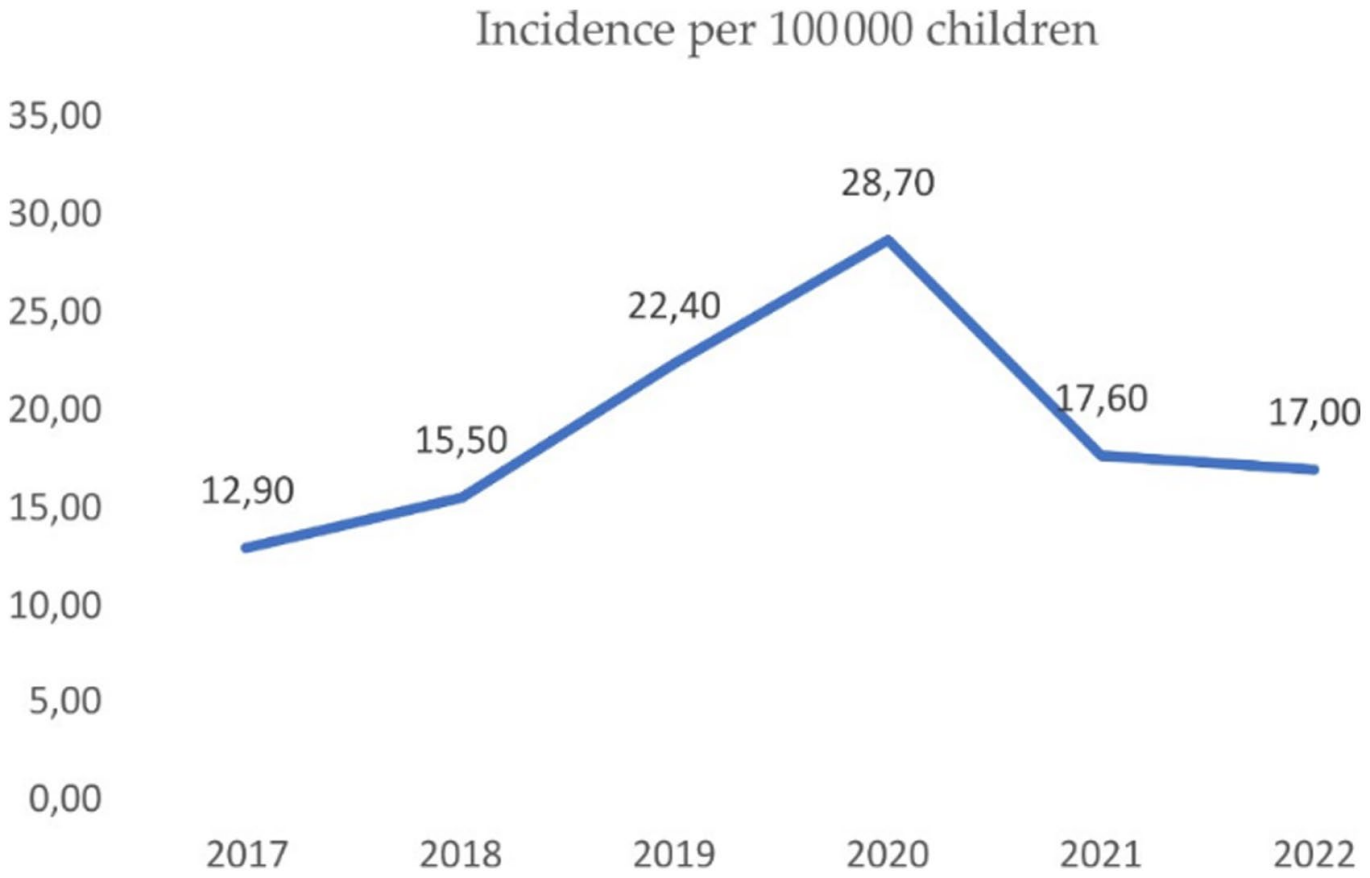
Incidence per 100,000 children in period of 2017–2022 in the Republic of Srpska, where we can see the linear trend till 2020, and decreasing in years after.

The Z-score for the incidence shows deviations from −2.41 to +4.18 in relation to the arithmetic mean, which clearly tells us that the incidence varied in this period. The year 2020 was the most interested one, because compared to 2019, the incidence increased by 28.13%, while in 2021, it decreased by 37% compared to 2020 (Figure 1).

### 3.1. Incidence by gender

Average incidence for boys in the period 2017–2022 was 19.5/100,000 of boys per year, while for girls it was 18.6/100,000. As we can see, in this period, boys have a higher incidence rate. However, if the standard arithmetic means of incidence for both groups are compared, it will be seen that both groups do not distinguish in average incidence rate (Levene’s test *F* = 3.376, *p* = 0.082). Also, the age at which type 1 diabetes was diagnosed does not differ by gender specific group (*t* = 0.288, *p* = 0.774).

### 3.2. Incidence by age groups

We divided the sample in the three specific age groups, 0–4 years of age, then 5–9 and the last group of 10–14 years of age, and calculated the incidence for each group separately per 100,000 children. The results are given in Table 2. We can see that the lowest number of newly diagnosed type 1 diabetes is in the age group of 0–4, only 21% in relation to the total number of cases, therefore the incidence in this age group is also the lowest, 12.5/100,000 children per year. The largest number of cases is in the group of children aged 10–14, 86 (47% in relation to the total number of newly detected cases) with the highest incidence of 26.1/100,000 children per year.

**Table 2:** Incidence of type 1 diabetes mellitus per 100,000 children per year by age groups in the period from 2017 to 2022.

Year	Number of diagnosed typed 1 diabetes by aged specific groups			Incidence per 100,000 children in aged specific groups		
	0–4	5–9	10–14	0–4	5–9	10–14
2017	4	6	11	7.6	11.3	19.5
2018	6	6	13	11.5	11.3	23.1
2019	8	12	16	15.3	22.7	28.6
2020	11	15	20	21.2	28.6	35.9
2021	6	9	13	11.7	17.3	23.5
2022	4	10	13	7.8	19.2	23.5
Total	39	58	86	12.5	18.4	26.1

Due to the small number of observed years (*n* = 6), we did not obtain statistically significant results in the incidence trend by age group.

### 3.3. Incidence depending on the state of admission before diagnosing the disease

In the past 6 years, a total of 73 cases of ketoacidosis or 39.9% of the total number of newly diagnosed type 1 diabetes mellitus were at the reception. Ketoacidosis, in relation to the total number of newly diagnosed cases of diabetes mellitus type 1, is the least present in the age group of 0–4 years of age (28.2%), while in the other two age groups it is present in approximately the same proportion. We can conclude that there is no statistically significant difference in the arithmetic means of groups of children who were admitted in a state of ketoacidosis or hyperglycemia in relation to age, because Levene’s test shows *F* = 1.26 and *p* = 0.723. The largest number of ketoacidosis was detected in 2020, where the incidence is the highest. There was no statistically significant correlation between gender and admission status, as Pearson’s Chi-square test was 1.327, *p* = 0.249 (Table 3).

**Table 3:** Number of ketoacidosis by age groups and years.

Year	Total number of newly diagnosed type 1 diabetes by specific age groups				Number of diagnosed ketoacidosis upon admission by specific age group (% of total number of newly diagnosed type 1 diabetes in that age group)			
	0–4	5–9	10–14	TOTAL	0–4	5–9	10–14	Total
2017	4	6	11	21	1 (25%)	3 (50%)	7 (63.6%)	11 (52.4%)
2018	6	6	13	25	3 (50%)	2 (33.3%)	8 (61.5%)	13 (52%)
2019	8	12	16	36	1 (12.5%)	2 (16.7%)	6 (37.5%)	9 (25%)
2020	11	15	20	46	4 (36.4%)	8 (53.3%)	6 (30%)	18 (39.1%)
2021	6	9	13	28	1 (16.7%)	5 (55.6%)	7 (53.8%)	13 (46.4%)
2022	4	10	13	27	1 (25.0%)	4 (40.0%)	4 (14.8%)	9 (33.3%)
Total	39	58	86	183	11 (28.2%)	24 (41.4%)	38 (44.2%)	73 (39.9%)

In further statistical analysis, we also took into account the distribution of ketoacidosis according to severity of it, based on blood pH and serum bicarbonate concentration (16). Mild ketoacidosis is defined as pH < 7.3 and serum bicarbonates HCO_3_^−^ < 15 mEg/L, medium is defined as pH < 7.2 and serum bicarbonates HCO_3_^−^ < 10 mEg/L, and severe as pH < 7.1 and serum bicarbonates HCO_3_^−^ < 5 mEg/L. Table 4 shows the distribution of ketoacidosis by severity for every year of observed period.

**Table 4:** Number of ketoacidosis by severity per year (% of ketoacidosis in the total number of newly diagnosed cases per year).

Year	Mild	Medium	Severe	Total
2017	3 (14.3%)	3 (14.3%)	5 (23.8%)	11 (52.4%)
2018	6 (24.0%)	3 (12.0%)	4 (16.0%)	3 (52.0%)
2019	5 (13.8%)	2 (5.6%)	2 (5.6%)	9 (25.0%)
2020	5 (10.7%)	7 (15.2%)	6 (13.0%)	18 (39.1%)
2021	2 (7.1%)	5 (17.9%)	6 (21.4%)	13 (46.4%)
2022	2 (7.4%)	2 (7.4%)	5 (18.5%)	9 (33.33%)
Total	3 (12.6%)	22 (12.0%)	28 (15.3%)	73 (39.9%)

Of the total number of ketoacidosis (73), which amounted to be the 39.9% of all cases of newly diagnosed diabetes mellitus type 1 in the period from 2017 to 2022 in the Republic of Srpska, the largest frequency was severe ketoacidosis (15.3% in total). The table shows that the percentage of ketoacidosis in total comparing to the number of all newly diagnosed type 1 diabetes, was higher in the period 2017–2019, or the pre-COVID-19 period. However, the most severe forms of ketoacidosis are significantly more prevalent in the COVID-19 period compared to the pre-COVID-19 period, while frequency of the mild forms of ketoacidosis have decreased in COVID-19 period compared to the total number of newly diagnosed type 1 diabetes.

Upon admission to hospitals in children diagnosed with ketoacidosis, the average value of HbA1c was 12.02%, while in children diagnosed only with hyperglycemia, HbA1c was 10.72%. Also, the average BMI was lower in children diagnosed with ketoacidosis (16.64 kg/m^2^), comparing to the children diagnosed only with hyperglycemia on admission (18.23 kg/m^2^). We also analyzed if there was dependence of the severity of ketoacidosis by gender (Pearson Chi-Square 0.082, *p* = 0.960), as well as the year of admission (Pearson Chi-Square 5.794, *p* = 0.670), however, we did not obtain valid statistical significance between these variables.

### 3.4. Number of newly diagnosed cases per month of the years

**Figure 2:**
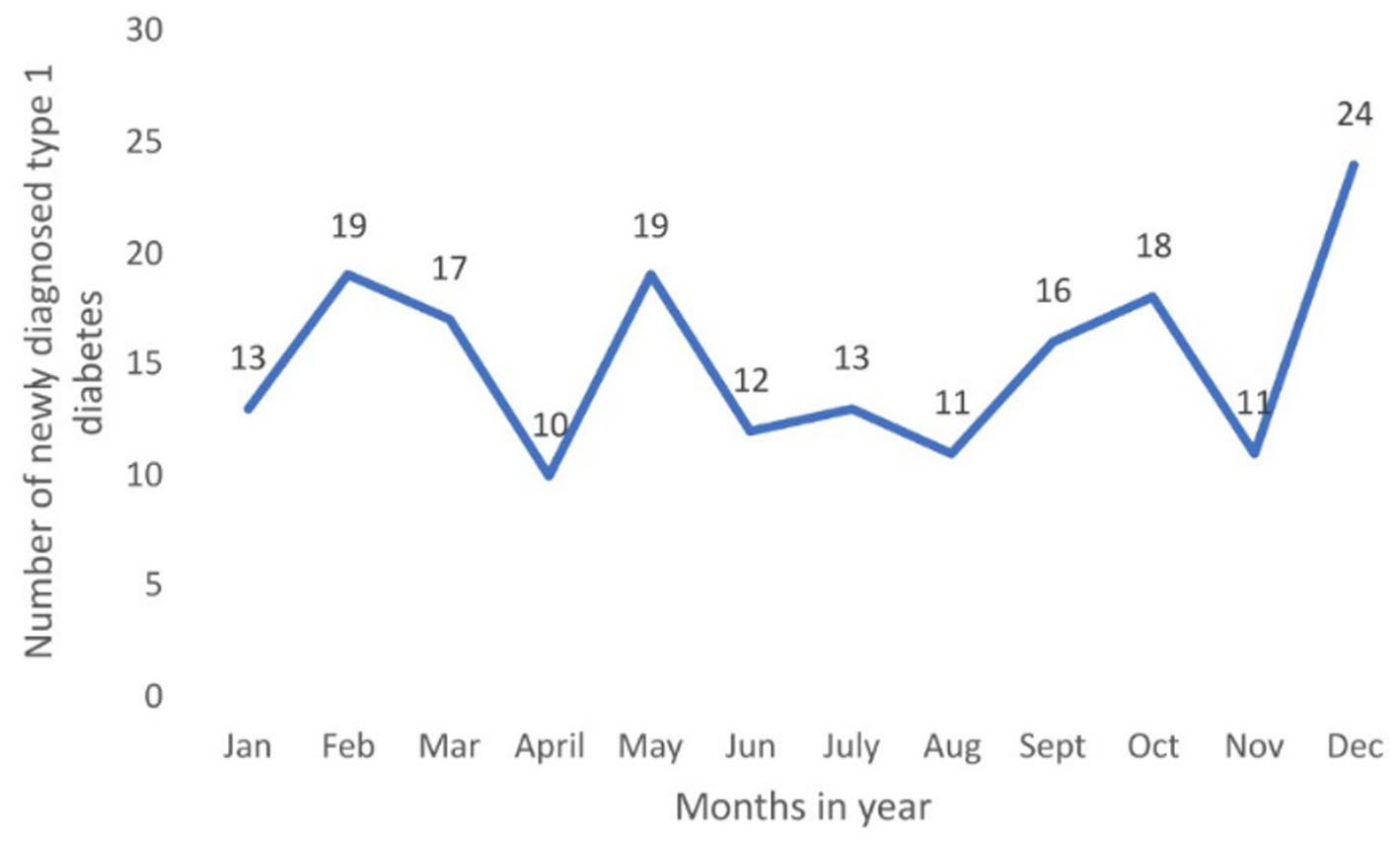
Number of newly diagnosed type 1 diabetes per month of diagnosis in period 2017–2022 in the Republic of Srpska.

In Figure 2, we see the distribution of newly diagnosed diabetes in children aged 0–14 according to the months of the year. It is noted that the largest number of newly diagnosed cases is in the winter months (December–March). In these periods, viral infections are commonly present, and it can be a trigger for the occurrence of diabetes in children (17), and this also coincides with the periods of the greatest restrictive measures during the COVID-19 pandemic in the Republic of Srpska.

## 4. Discussion

According to the results obtained in this study, we see that the incidence of diabetes mellitus type 1 in the period 2017–2022 increased compared to the period from 2001–2016, from 11/100,000 to 19.5/100,000 children aged 0–14 per year. The linear trend was observed in the period 2001–2016 (*r* = 0.71, *p* ≤ 0.002) (4). Due to the small number of observed years (*n* = 6) in the period from 2017 to 2022, we did not obtain statistically significant results that would confirm a linear trend in this period (*r* = 0.31, *p* ≤ 0.54). Combining the results from these two periods would probably yield statistically significant linear trends and this could be a topic for future research. The IDF estimates that in Bosnia and Herzegovina in 2021 the incidence rate is less than 10/100,000 children aged 0–14 (9), which does not correlate with our findings. This discrepancy is explained by the complicated state structure of Bosnia and Herzegovina, and the absence of a single national register of diabetes patients in the country, which would collect data in one place and facilitate planning and prevention of this disease (18). The annual growth of incidence is in line with study models that predict that by 2040, the number of people suffering from diabetes mellitus type 1 will increase by 49% compared to 2020 (19). If we follow these trends in Bosnia and Herzegovina, there will be the increase not only in the incidence of diabetes but also in the number of ketoacidosis that will manifest when new cases are diagnosed. Currently, with an incidence of 19.5/100,000 children per year for the age group of 0–14 years in the period 2017–2022 we entered the group of high-risk countries for diabetes type 1 according to the WHO classification.

In comparison with available data from neighboring countries and regions, the incidence of type 1 diabetes mellitus in the Republic of Srpska is higher than in Serbia (14.3/100,000 for the period 2007–2017), Montenegro (19.2/100,000 for the period 2016–2020), Croatia (17.2/100,000 for the period 2004–2012), Vojvodina (11.9/100,000 for the period 2017–2021) (20–23). Our finding shows that the highest incidence of 28.7/100,000 was recorded in the first year of the global COVID-19 pandemic, i.e., in 2020, and compared to 2019, it increased by 28.13%. Growth trend like this, and even greater, can be also observed in the available data from the countries of the region. The recorded growth can be interpreted as the influence of external factors such as fear of the unknown, limited movement, reduction of contacts, increased intake of sweets in conditions of isolation, home learning. Other possible explanation is that the exposure to COVID-19 accelerate the onset of type 1 diabetes mellitus in children (11). The acceleration of the onset leads to more newly diagnosed patients in 2020 and fewer in 2021, and the fact that both of these years deviate from the linear trend seen in the years before 2020 suggests that COVID-19 can accelerate the onset of the type 1 diabetes. Also, this explains the decrease in 2021. Besides that, the decrease in incidence in 2021 can also be explained by the fact that the COVID-19 restrictive measures in this year were not so strict and the population got used to it. The children returned to their school duties, but still wore masks and thus protected themselves not only from COVID-19, but also from other common viruses that can be a trigger for a newly onset of type 1 diabetes (15). Another possible explanation of the decrease of newly diagnosed type one diabetes in 2021 is that in 2020 people started using more vitamin and mineral supplements and boosted their immune system. Effect of that supplement usage presented in 2021 in full potential. Connection between using the vitamins supplement during the COVID-19 pandemic and the onset of type one diabetes can be subject of some other research.

Out of the total number of patients, 39.9% were admitted with symptoms of ketoacidosis in this period as our results show. In America, new cases of ketoacidosis increased from 31 to 51% from 2008 to 2017, in Canada, the prevalence increased from 18.6 to 25.6% (12, 24). The percentage of children and adolescents presenting in a state of ketoacidosis at the time of diagnosis also varies between countries and depends on the average income. According to predictions, in poorer countries (in low-income countries) new patients with ketoacidosis should decrease from 80% in 1990 to 40% in 2050, while in highly developed countries the number of children should decrease to 20–30% in 2050 (3). Children may be misdiagnosed as newly diagnosed diabetes mellitus type 1 due to initial non-specific symptoms, which happened in as many as 24% of cases in America. In poorer countries, the percentage of wrong diagnosis is even higher. The highest percentage of newly diagnosed cases of ketoacidosis is expected in Africa and Asia, that is, countries with low incomes and poor countries (19).

Most of the published studies, which are still limited, record the increase in the incidence of type 1 diabetes and ketoacidosis during the pandemic period. We investigated the period before, during and after COVID-19 global pandemic and recorded a significant increase in the number of patients in the year of 2020 compared to previous years, and in 2021 we recorded a significant decrease in new cases. Also, a higher percentage of severe ketoacidosis was observed in the pandemic years compared to the previous period. Regular check-ups at physician were not possible due to the COVID-19 pandemic, so the parents probably overlooked the first symptoms of diabetes in children, and that could lead to increase number of severe ketoacidosis. In our country only one child had COVID-19 infection together with newly diagnosed type 1 diabetes. In that case COVID-19 was diagnosed on routine check-up after the patient admitted to hospital with the symptoms of severe ketoacidosis. In this patient the mild symptoms of COVID-19 infection did not require the steroids therapy. Since the serological assay for COVID-19 antibodies were not conducted on all newly diagnosed patient with type one diabetes, we do not have enough data that could connect the steroids usage as part of COVID-19 treatment and onset of type one diabetes. In America, only 4 children out of 187 patients (2.1%) had a COVID-19 infection at the time of illness. The results of this study are the most similar to ours, as the five-year retrospective incidence of type 1 diabetes before COVID-19 was shown, and it was showing the linear growth This growth continued during the COVID-19 pandemic, where the number of patients in a state of ketoacidosis also increased (25).

The results of the study in Canada did not show an increase in incidence of type 1 diabetes during the COVID-19 period, compared to pre-COVID-19 period. In Canada, a significant increase in the frequency of ketoacidosis among new patients was recorded in the pandemic compared to the pre-pandemic period (68.2% compared to 45.6% in the pre-pandemic period with *p* < 0.001) (13). The largest Polish National Diabetes Center did not record a statistically significant increase in 2020 compared to 2019, but a significant increase in diabetic ketoacidosis was recorded (35.2–47.53%, *p* < 0.05), which was accompanied by a significant increase in average HbA1c and pH values (26).

It will be interesting to observe the incidence in the next few years, in which we will get confirmation of whether COVID-19 accelerates the onset of type 1 diabetes. Our results show that this global pandemic has impacted the incidence of type 1 diabetes as well as the frequencies of ketoacidosis in our region. The exact mechanism of this influence has yet to be investigated.

## 5. Conclusion

The trend of increasing incidence of diabetes in the world is a well-known fact. However, what is worrying are prognostic data, according to which there will be even greater growth by 2050. In Republic of Srpska, as part of Bosnia and Herzegovina which is middle income country (27), it is expected that this increase should be higher than the European average, while the results of this study for the period 2017–2022 put us in the European average. Of particular concern is the fact that the average rate of increase in the incidence of type 1 diabetes in our region has significantly increased compared to previous observed periods in all age groups.

One of the ways of preventing diabetic ketoacidosis, which can be applied in all countries, is the education of the entire population on recognizing the symptoms of diabetes. This is especially important for any state of emergences such as the pandemic period, and therefore efforts should be made at the national level to prevent the occurrence of diabetic ketoacidosis. In case of any future pandemic, we should expect the higher number of newly diagnosed type 1 diabetes, and therefore the increased frequencies of ketoacidosis that could additionally burden the health systems, which are vulnerable in periods like this. So, findings of our research should warn us to have action plans in periods like this. Also, the prevention of the increase in the incidence of type 1 diabetes mellitus should be reflected in changing lifestyle habits, improving the quality of life through healthy eating and increased physical activity among young people.

## Data availability statement

The raw data supporting the conclusions of this article will be made available by the authors, without undue reservation.

## Ethics statement

The studies involving humans were approved by the Ethics Committee of University Clinical Center of the Republic of Srpska (protocol number: 01-19-82-2/23 from March 22, 2023). The studies were conducted in accordance with the local legislation and institutional requirements. Written informed consent for participation was not required from the participants or the participants’ legal guardians/next of kin in accordance with the national legislation and institutional requirements.

## Author contributions

GB-R: Conceptualization, Supervision, Writing – original draft, Writing – review and editing, Investigation. VM: Formal analysis, Funding acquisition, Validation, Writing – review and editing. OL: Data curation, Investigation, Methodology, Writing – review and editing.

## Funding

The author(s) declare that no financial support was received for the research, authorship, and/or publication of this article.

## Conflict of interest

The authors declare that the study was conducted in the absence of any commercial or financial relationships that could be construed as a potential conflict of interest.

## Publisher’s note

All claims expressed in this article are solely those of the authors and do not necessarily represent those of their affiliated organizations, or those of the publisher, the editors and the reviewers. Any product that may be evaluated in this article, or claim that may be made by its manufacturer, is not guaranteed or endorsed by the publisher.
